# Cystine Renal Calculi: New Aspects Related to Their Formation and Development

**DOI:** 10.3390/jcm13102837

**Published:** 2024-05-11

**Authors:** Felix Grases, Francisca Tomàs Nadal, Francesca Julià Florit, Antonia Costa-Bauza

**Affiliations:** Laboratory of Renal Lithiasis Research, University Institute of Health Sciences Research (IUNICS-IdISBa), University of Balearic Islands, 07122 Palma de Mallorca, Spain; fgrases@uib.es (F.G.); xiscatn@gmail.com (F.T.N.); f.julia@uib.es (F.J.F.)

**Keywords:** cystine, renal calculi, thiol-binding compounds, crystallization inhibitors

## Abstract

**Background**: Crystallization experiments of renal-calculi-forming compounds (calcium oxalate, calcium phosphates, uric acid) are normally performed by monitoring these processes during periods of time similar to the residence of urine inside the kidney. Nevertheless, cystine requires high supersaturation for its crystallization, and most experiments last for longer periods. It must be considered that at high supersaturation, the inhibitors of crystalline development have poor effects. **Methods**: The induction time of crystallization (t_i_) of cystine in experimental conditions similar to those of the formation of cystine renal calculi and the effect of different cystine-binding thiol agents was determined through turbidimetric measurements. We also studied the macro- and microstructure of 30 cystine kidney stones through stereoscopic microscopy and scanning electron microscopy. **Results**: Under the studied conditions, the t_i_ in absence of crystallization inhibitors was 15 min, and the presence of 9 mM of penicillamine, tiopronin, or N-acetylcysteine totally inhibited crystallization, as their effects relate to the formation of complexes with cystine, although N-acetylcysteine also delayed cystine crystalline development and modified cystine crystal morphology. Cystine stones have traditionally been classified as smooth and rough. The study of their structure shows that all of them begin their formation from a few crystals that generate a compact radial structure. Their subsequent growth, depending on the renal cavity where they are located, gives rise to the rough structure in the form of large blocks of cystine crystals or the smooth structure with small crystals. **Conclusions**: To prevent the development of cystine renal stones, the formation of small crystals must be avoided by reducing urinary cystine supersaturation, with N-acetylcysteine being the most effective among the studied cystine-binding thiol agents. Also, the removal of cystine crystals through increased water intake and physical activity can be a very important preventive measure.

## 1. Introduction

Urolithiasis is a frequent urinary system disease with a medium prevalence of around 10% of the population [1], and this prevalence is increasing worldwide. Socio-economic status is also a fundamental risk factor for this disease [2].

Cystine kidney stones are the most common type of renal calculi in patients with monogenic nephrolithiasis, and they account for about 1% of all renal calculi and 4 to 8% of renal calculi in children [3,4,5]. These stones form when L-cystine (which has low aqueous solubility) crystallizes in the urine. Although L-cystine stones are much less common than calcium oxalate stones, L-cystine stones are larger, occur in younger individuals, have a higher rate of recurrence, and are frequently associated with chronic kidney disease (CKD) [6].

Genetic studies have indicated that there are two major types of cystinuria. Type A is due to mutations in both copies of the SLC3A1 gene (genotype AA) and type B is due to mutations in both copies of the SLC7A9 gene (genotype BB) [7,8]. Fewer than 2% of patients with a mutation in only one allele (A0 or B0) develop cystinuria. Nevertheless, the severity of the clinical phenotype cannot be explained exclusively by excretion of cystine, and it is necessary to identify other genetic and environmental factors that affect this disease.

Our review of studies that examined cystine crystallization in aqueous media showed that these studies monitored crystallization kinetics in a very different way from crystallization studies that were performed for other uroliths, such as calcium oxalate, hydroxyapatite, brushite, and uric acid. Typically, crystallization experiments are performed by monitoring the process during periods of time similar to the residence times of urine inside the kidney (minutes). Nevertheless, cystine requires high supersaturation for its crystallization, and the experimental designs found in the literature to achieve it consist of the slow evaporation of the solvent [9], the cooling of the solution from a very high initial temperature [10], or the acidification of a neutral or basic cystine solution in which cystine is more soluble [11]. Obviously, the procedures used for both evaporation and cooling require longer periods of time (hours or days) to induce crystallization [12,13,14]. Because of this, monitoring of the crystallization kinetics is not usually possible. Because both homogeneous and heterogeneous nucleation of cystine crystals is difficult, a very highly supersaturated solution is necessary to achieve crystallization in urine.

Studies that examined large collections of cystine stones showed that these stones can be classified into two large groups: rough calculi and smooth calculi [15,16]. The rough cystine stones consist of well-formed blocks of hexagonal crystals, and the smooth cystine stones are formed through the intergrowth of small cystine crystals. This classification, apart from its use in establishing the mechanism of stone formation, also has practical importance because smooth stones are less sensitive to fragmentation by extracorporeal shock wave lithotripsy.

In this paper, we first present a kinetic study of the crystallization of cystine in saline solution, with and without cystine-binding thiol agents, to assess factors that may affect the induction time of cystine crystallization in the urine. Then, we performed an extensive study of cystine kidney stones that were formed by different patients with cystinuria and characterized their structure and possible mechanism of formation.

## 2. Materials and Methods

### 2.1. Crystallization Studies

#### 2.1.1. Reagents and Solutions

Cystine, sodium chloride, sodium cyanide, sodium nitroprusside, tiopronin, N-acetylcysteine, and penicillamine were purchased from Sigma-Aldrich (St. Louis, MO, USA). All chemicals were of analytical/reagent grade and dissolved in ultra-pure deionized water from a Milli-Q system. A cystine stock solution (700 g/L, 2.9 mM) was prepared daily by dissolving 0.35 g of cystine and 8.33 g of NaCl in 0.5 L of ultra-pure deionized water, followed by the addition of 3 M NaOH to achieve a final pH of 8.0.

#### 2.1.2. Turbidimetric Assay

A new kinetic turbidimetric assay was developed and used to determine the effect of selected compounds on cystine crystallization in aqueous medium that contained 700 mg/L (2.9 mM) cystine and 0.28 M NaCl at a pH of 3.5 to 3.7. The cystine concentration and pH value were optimized in order to obtain cystine crystallization induction time values that were within the range of the residence time of urine in the urinary tract. Thus, they should not be excessively short, as, under these conditions the effectiveness of the inhibitors is considerably reduced, nor excessively long, as this causes low reproducibility. The turbidimetric system consisted of a photometer (AvaSpec-ULS2048CL-EVO, Avantes, Apeldoorn, The Netherlands) that had a fiber optic light-guide measuring cell, which was attached to a light path reflector (2 × 10 mm). This instrument was operated in kinetic mode, and absorbance measurements were integrated from 400 to 600 nm. Crystallization was assessed at a constant temperature (25 °C) with mixing using a magnetic stir bar (400 rpm).

A stock solution of cystine (100 mL) was added to a crystallization flask, followed by the addition of 1 mL of water. Then, 0.3 mL of 1 M HCl was added to achieve a pH of 3.5 to 3.7 and to induce cystine supersaturation (SS = C/C_eq_) of 4.8 based on the known equilibrium solubility (C_eq_) of 0.6 mM at 25 °C in 0.3 M NaCl and a pH of 4 to 6 [17]. Under these conditions, cystine crystallization had a relatively short induction time (10–20 min without an inhibitor). Absorbance was recorded continuously to monitor crystal formation.

**Figure 1:**
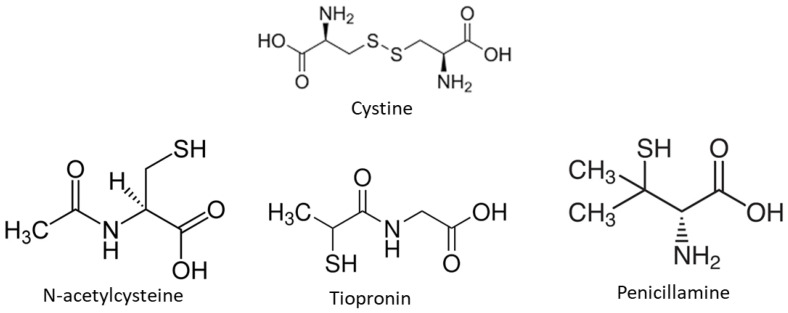
Chemical structures of cystine, N-acetylcysteine, tiopronin, and penicillamine.

Two crystallization inhibitors (penicillamine and tiopronin) were selected among the compounds currently used for the treatment of cystinuric stone-forming patients [18], and N-acetylcysteine was also tested because its effectiveness in the treatment of cystinuric stone-forming patients was previously reported [19,20], as well as its effectiveness in the dissolution of cystine stones [21]. In fact, it is recommended for percutaneous chemolysis [18]. The studied substances (shown in Figure 1) were assayed at concentrations of 0.14 to 9 mM. These concentrations were achieved by adding an appropriate volume of a 0.9 M stock solution of each compound to the crystallization mixture, with a corresponding reduction in the volume of water. The induction time (t_i_) was defined as the time when the absorbance first began to increase due to crystal formation. Each experiment was performed in triplicate, and mean values were reported.

#### 2.1.3. Cystine Determination

The effects of these different compounds on cystine solubility were also evaluated at 24 h after the induction of crystallization (t_i_) by measuring the concentration of cystine remaining in the solution. In these experiments, the concentration of cystine remaining in the supernatant after 24 h was determined using a 96-well plate with an adaptation of an established cyanide–nitroprusside colorimetric method [22]. Prior to these measurements, the potential effect of each compound on interference with the determination of cystine was determined, because free sulfhydryl groups (or disulfide bonds after reduction by cyanide) react with nitroprusside in an alkaline solution to yield a red complex. Thus, the absorbance of each measurement was corrected based on the presence of the compound at the specific concentration. Notably, products of cystine transformation due to a reaction with cystine-binding agents can also lead to a positive reaction in the cyanide–nitroprusside colorimetric method.

Cystine standard solutions (20–400 mg/L) were prepared through appropriate dilution of a cystine stock solution. The assays were performed in triplicate in 96-well plates, and each well contained 80 µL of a standard (or a 50% or 80% diluted sample) and 150 µL of sodium phosphate buffer at pH 7.3 (PBS) with 0.01 M phosphate, 0.15 M NaCl, and 90 µL of 10% (*w*/*v*) sodium cyanide. After addition of sodium cyanide, the sample was incubated at room temperature for 10 min. Color development was induced by the addition of 30 µL of a 20% (*w*/*v*) sodium nitroprusside solution. The absorbance of each well was then measured at 521 nm within 1 min using a Microplate spectrophotometer (Powerwave XS, Biotek Instruments, Inc., Winoosk, VT, USA).

#### 2.1.4. Cystine Crystals’ Morphology

The effects of different compounds on cystine crystallization were also qualitatively evaluated using scanning electron microscopy (SEM; TM4000 Plus II, Hitachi, Tokyo, Japan). For these measurements, a pipette was first used to collect crystals from the bottom of the well. Then, the crystals were placed on a sample holder, fixed with adhesive conductive tape, and then dried using paper to absorb any remaining liquid.

#### 2.1.5. Statistics

Comparisons of induction time between absence of additives and different concentrations of tested compounds were performed using a one-way ANOVA test. A two-tailed *p*-value less than 0.05 was considered statistically significant. Statistical analyses were performed using SPSS version 23.0 (SPSS Inc., Chicago, IL, USA).

### 2.2. Cystine Renal Stones

Thirty-five cystine kidney stones from 6 different patients with monogenic nephrolithiasis were also studied. These calculi were identified through morpho-compositional analysis. First, the stones were observed using stereoscopic microscopy (MOTIC SMZ-161, MoticEurope, Barcelona, Spain) to identify the most significant areas for subsequent analysis, which consisted of infrared spectroscopy (Bruker Hyperion IR, Bruker, Berlin, Germany), imaging with scanning electron microscopy (SEM; TM4000 Plus II, Hitachi, Tokyo, Japan), and energy-dispersive X-ray microanalysis (Quantax 75 EDS microanalyzer, Bruker, Berlin, Germany).

## 3. Results

### 3.1. Crystallization Studies

**Figure 2:**
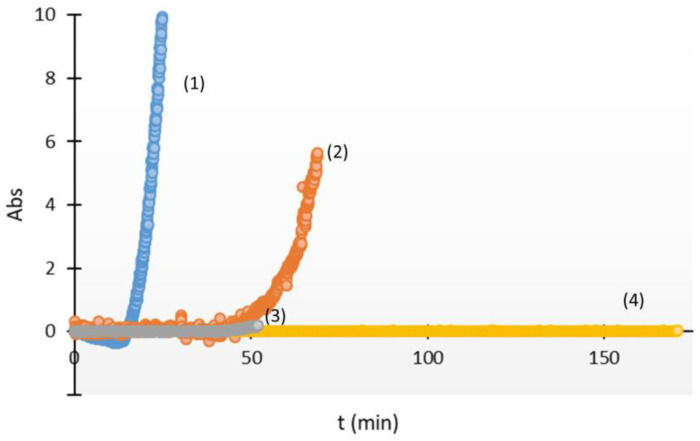
Kinetics of in vitro cystine crystallization in absence of additives (1) and in presence of 0.125 mM of penicillamine (2), 0.56 mM of tiopronin (3), and 0.28 mM of N-acetylcysteine (4).

The method used for the turbidimetric assay of the in vitro crystallization of cystine consisted of generating a supersaturated solution through acidification of a solution that contained 700 mg/L of cystine. Under these conditions, the t_i_ in absence of crystallization inhibitors was 15 min (n = 9, SD = 3.7, Figure 2). The addition of either penicillamine, tiopronin, or N-acetylcysteine at a concentration of 9 mM totally inhibited crystallization under these conditions. This highest tested concentration was within the range of concentrations studied previously [23].

**Figure 3:**
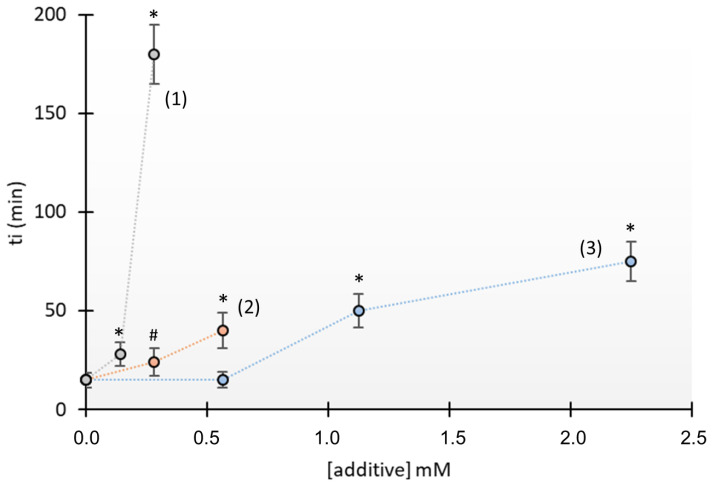
Effect of N-acetylcysteine (1), tiopronin (2), and penicillamine (3) on the induction time (t_i_) for in vitro cystine crystallization. Induction times were obtained as in Figure 2, and values are the means ± SD of three replicates (except in the absence of additives, which includes 9 replicates). # *p* < 0.05 vs. absence of additives, * *p* < 0.005 vs. absence of additives.

We then examined the effects of lower concentrations of these compounds on the t_i_ (shown in Figure 2 and Figure 3). The results show that N-acetylcysteine had the strongest inhibition, and that an effect began to manifest at 0.14 mM. The concentrations needed for penicillamine (0.28 mM) and tiopronin (1.12 mM) were both greater (Figure 3).

**Figure 4:**
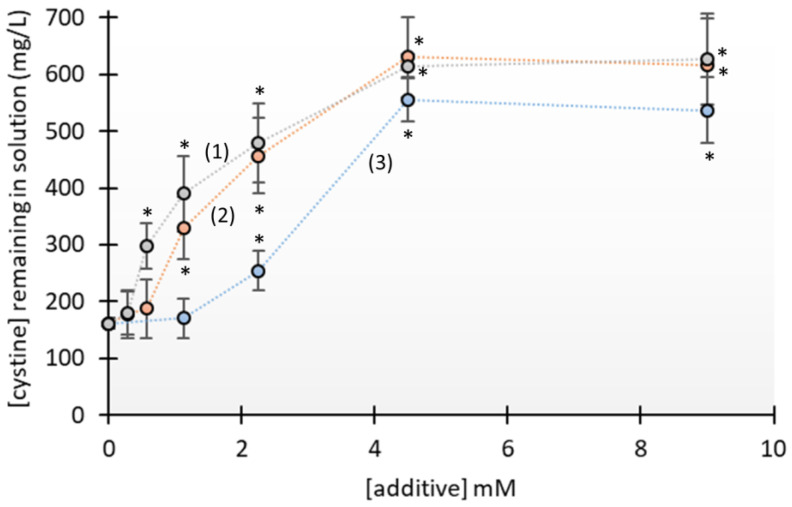
Concentration of cystine remaining in solution after 24 h in vitro with different levels of N-acetylcysteine (1), tiopronin (2), or penicillamine (3). All values are the means ± SD of three replicates (except in the absence of additives, which includes 9 replicates). * *p* < 0,005 vs. absence of additives.

We also used the cyanide–nitroprusside assay to measure the concentration of cystine that remained in the solution after 24 h when the initial cystine concentration was 2.9 mM (700 mg/L), with or without addition of these different inhibitors. Because penicillamine and N-acetylcysteine also react to nitroprusside, the absorbance increase due to their presence at each concentration was subtracted from the measured absorbance; tiopronin did not react with the cyanide–nitroprusside in these conditions. The results show that each of the three compounds increased the apparent solubility of cystine (see Figure 4). In particular, the concentration of cystine remaining in the solution after 24 h in the absence of additives (Ceq) was 160 mg/L, which is close to the previously reported solubility of cystine under similar conditions (0.6 mM, 144 mg/L) [17]. For experiments performed with 4 mM or greater levels of N-acetylcysteine, tiopronin, or penicillamine, the concentration of cystine remaining in the solution after 24 h was close to its initial concentration (see Figure 4), indicating no cystine crystallization. However, N-acetylcysteine and tiopronin were more effective at lower concentrations than penicillamine.

**Figure 5:**
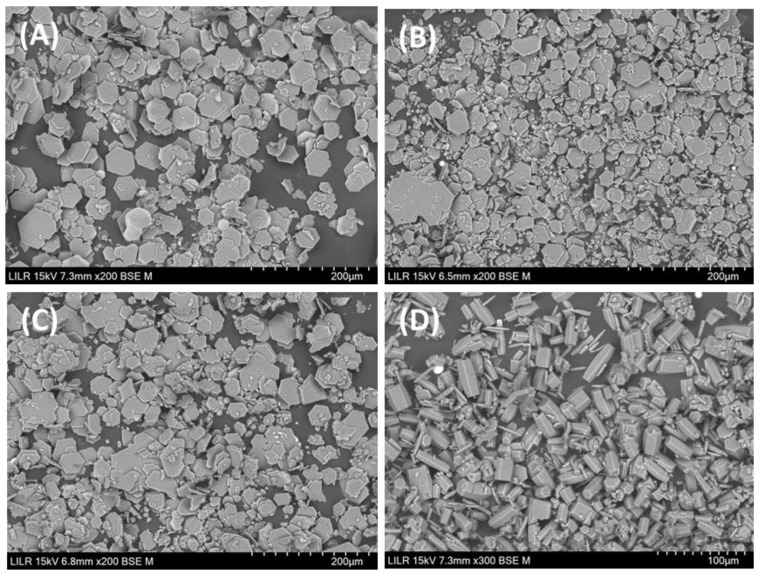
Representative scanning electron microscopy images of cystine crystals that formed in vitro in the absence of additives (**A**) or with 2.25 mM of penicillamine (**B**), 1.125 mM of tiopronin (**C**), or 0.28 mM of N-acetylcysteine (**D**).

We then examined the effects of these different crystallization inhibitors on the morphology of the cystine crystals. Scanning electron microscopy showed that N-acetylcysteine was the only compound that altered the morphology of crystals, and these crystals had a polyhedral shape (shown in Figure 5D). The crystals in the other three treatments were hexagonal (see Figure 5A–C). Notably, N-acetylcysteine affected the morphology of cystine crystals at a concentration of only 0.28 mM (shown in Figure 5); the same concentration of penicillamine or tiopronin had no apparent effects (see Figure 3).

### 3.2. Morphology of Cystine Stones

We then examined 30 cystine kidney stones that were collected from six patients, each of whom passed 4 to 11 stones (see Table 1). Our results showed that a single patient can generate rough stones and smooth stones, and that almost all of the stones had hexagonal crystals, which form more slowly and are the thermodynamically stable crystalline form of cystine. Polyhedral crystals (shown in Figure 5D), which are the kinetically favorable form and generate at a higher rate, were not observed in cystine kidney stones.

**Figure 6:**
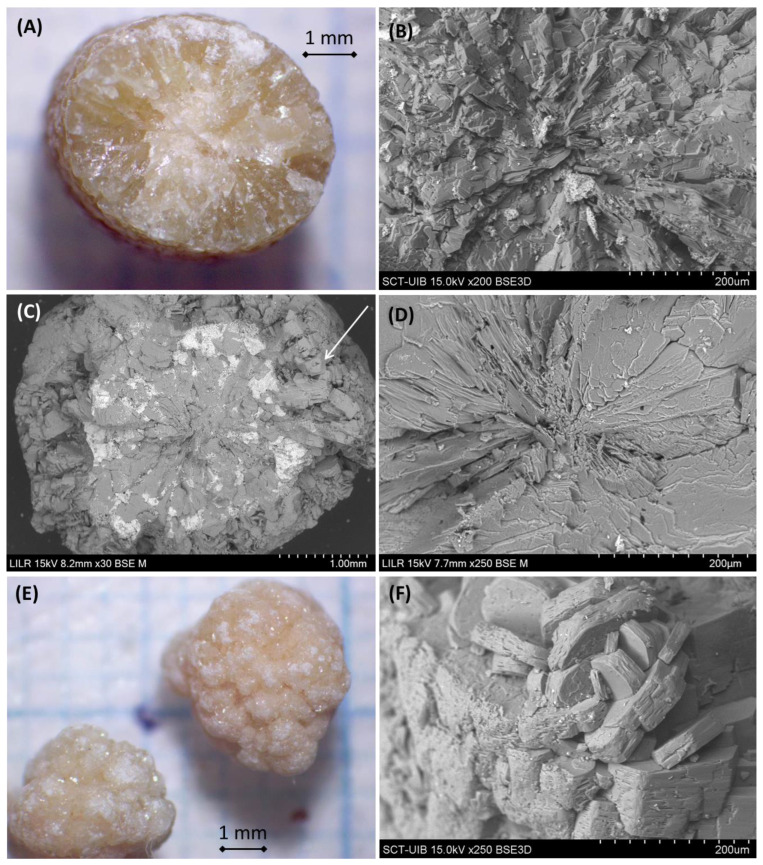
Representative images of cystine stones. (**A**) Stereoscopic microscopy of a section of a smooth stone, showing its radial structure. (**B**) Scanning electron microscopy image of the center of the stone in A, showing small crystals that constituted the origin. (**C**) Scanning electron microscopy image of a section of a rough cystine stone, showing that only the center has a radial structure, which is surrounded by large blocks of cystine crystals (see arrow). (**D**) Scanning electron microscopy image of the center of the stone in (**C**), showing that its origin was the same as that for the smooth stone. (**E**) Stereoscopic microscopy (external image) of rough stones. (**F**) Scanning electron microscopy image of the external part of a rough stone, showing disordered growth of large blocks of cystine crystals.

The smooth stones apparently developed in narrow cavities in which there was low urodynamic efficiency and limited fluid entry, conditions that favor the formation of hexagonal cystine crystals; these stones also contain small amounts of other materials, such as organic matter and calcium phosphates. This accumulation led to the growth of columnar and intergrowth cystine crystals with a compact structure, constituting the body of the smooth stones (shown in Figure 6A,B). It is interesting that the upper area of these stones had small, ordered cystine crystals.

The rough stones had surfaces with large hexagonal cystine crystals (see Figure 6C,E,F) and consisted of large blocks of primary aggregates of hexagonal cystine crystals. These were generated around small deposits of organic matter and/or calcium phosphate deposits. It is important to note that these rough stones were practically identical to smooth stones at the zone of the beginning of formation. Thus, a deposit of small cystine crystals induces the formation of large cystine crystals in a columnar arrangement (see Figure 6D), and then large blocks of cystine crystals (see Figure 6C) subsequently develop and generate the rough morphology (see Figure 6E). These structures were apparently generated in more open cavities that had greater contact with urine and higher urodynamic efficiency.

These results suggest that all cystine stones begin to form through a similar mechanism in which an initial deposit of small amounts of cystine crystals and other materials (organic matter, hydroxyapatite) induces the formation a central area with columnar crystals. These initial crystals subsequently develop into large blocks of cystine crystals when they are in open areas with efficient urodynamics and into compact areas of small cystine crystals when they are in more closed areas with poor urodynamics.

## 4. Discussion

Our study of cystine kidney stones showed that these stones were always generated in kidney cavities, and there was no embedding of these stones in kidney tissue. According to previous studies [15,16], cystine stones can be classified as rough stones or smooth stones. It is important to note that both types of stones can occur in the same patient, and both have areas containing small amounts of small cystine crystals that may also sometimes include organic matter or hydroxyapatite. These areas at the stone’s core seem to correspond to the onset of stone formation. If the stone forms in a large cavity, this initial core can grow without restrictions, and some crystals undergo epitaxy and induce the development of others that ultimately generate lobes. These lobes stop developing when they meet with other nearby areas, and this culminates in the formation of a rough cystine renal calculus. However, if the stone is generated in a narrow cavity of a certain depth, the growth of cystine crystals in the core develops into columnar crystals with a palisade structure (shown in Figure 6A). This growth leads to a more compact and smooth structure. Therefore, the shape of the cavity in which a cystine stone first develops determines its final morphology. Obviously, a more compact and smooth stone is more difficult to break using physical methods. Our finding that all cystine stones begin from a small number of small cystine crystals raises an important issue regarding methods that can prevent the formation of these stones. In particular, if the initial crystals could be eliminated before they start to grow, this could prevent stone formation. The elimination of these initial crystals could be achieved through interventions that remove them from renal cavities, and this can be induced through high liquid flow (high water intake) [24] combined with intense movement (physical activity).

Previous in vitro cystine crystallization studies demonstrated that these crystals do not easily form in a liquid medium whose composition is similar to urine and instead require a high supersaturation of cystine [12,13,14]. This seems to indicate that these crystals form through homogeneous nucleation. In agreement, no previous studies have reported the presence of effective heterogeneous nucleants for the formation of cystine crystals. It is important to consider that the effect of inhibitors of crystalline development is even greater at a lower level of supersaturation, when the growth of crystals occurs primarily through dislocations (based on second-order parabolic growth). In these cases, blocking the different growth points determines inhibitory activity. However, for a very high supersaturation, crystal growth occurs mainly through surface nucleation (based on exponential growth of an order higher than two), and the effect of an inhibitor will be decreased. Interestingly, we found that at very low concentrations of N-acetylcysteine (0.28 mM), the cystine crystals had a polyhedral morphology (shown in Figure 5D), the kinetically favored form. This result may be because the interaction of N-acetylcysteine with the crystals destabilizes the hexagonal shape, the thermodynamically favored form. This shows that these inhibitors also interact with the crystals after their generation, as occurs in cases of crystallization processes at low supersaturation (growth through crystalline dislocations). In fact, the inhibitors we studied had two basic mechanisms: they interacted with cystine in solution and they interacted with the cystine crystals. In the former mechanism, these inhibitors generate new substances that reduce supersaturation because they are thiol-binding compounds that react with cystine and form other soluble disulfides [20]. However, interaction with the cystine crystal will only exhibit notable effects when there is a low level of supersaturation, when the crystal grows through dislocation (not through surface nucleation). Thus, although a low concentration (0.28 mM) of N-acetylcysteine affected cystine crystallization and it also modified cystine crystal morphology, penicillamine and tiopronin did not have significant effects at this concentration. It should also be noted that most other crystallization inhibitors of kidney-stone-forming compounds also exhibit both mechanisms, in that they reduce supersaturation and inhibit crystalline growth after induction. The concentration of the inhibitor and the concentration of the solute determine which mechanism of inhibition occurs. A typical example is calcium and citrate during the formation of calcium oxalate crystals [25]. Calcium forms soluble complexes with citrate (reducing the supersaturation of calcium oxalate) and also binds to calcium oxalate crystals at specific points, leading to decreased or disrupted crystal growth.

Therefore, for a solute that requires high supersaturation to achieve crystallization, inhibitors of crystalline development will only have minor effects. In these cases, alternative interventions that decrease supersaturation should be considered. This could be achieved by promoting the formation of complexes, chemical transformation of the solute (e.g., through a redox reaction), or stimulating the formation of clusters. In the case of cystine, these effects can be achieved with some of the substances examined in the present study. For example, D-penicillamine forms a soluble complex with cystine that increases its solubility by about 50-fold [18], and a 9 mM concentration of D-penicillamine prevented cystine precipitation for at least 24 h under conditions of high cystine supersaturation (SS = 4.8, corresponding to 2.9 mM cystine at pH = 3.5). Captopril has a similar effect, in that it induces the formation of a thiol–cysteine mixed disulfide that is 200-fold more soluble than cystine [26]. Unfortunately, both of these products have severe adverse effects in vivo [26,27,28]. N-acetylcysteine is also very effective in preventing the crystallization of cystine [20,29], but because it also increases the excretion of cystine it is not recommended for prevention of cystinuria [29]. Nevertheless, considering our finding that a relatively low concentration of N-acetylcysteine (0.28 mM) was effective in the presence of 2.9 mM of cystine, consistent with previous findings [20], we suggest reconsidering the use of a low dose of N-acetylcysteine for the treatment of cystinuria. It would be necessary to carry out a study of the relationship between the consumption of low doses of N-acetylcysteine and the urinary excretion of cystine in cystinuric patients. In fact, an important limitation of the present study is that this relationship is not known, so the use of N-acetylcysteine cannot be recommended at this time for the treatment of cystinuric patients. It is likely that a concentration that does not significantly increase cystinuria will not lead to other significant secondary effects. In fact, cystine renal stones have been successfully dissolved with alkaline solutions of N-acetylcysteine using transurethral catheters [21].

## 5. Conclusions

Solutes that are resistant to crystallization only form stones in the urine when they reach a very high supersaturation, and inhibitors of crystalline development therefore have little effect on the development of crystals from these solutes. In these situations, other compounds must be used to reduce the supersaturation of the solute by increasing the formation of soluble complexes, by inducing the formation of stable soluble clusters, or by chemically transforming the solute through some type of reaction, such as a redox process. In this sense, it is important to identify new molecules with potential application to humans that can interact with cystine, thus leading to the formation of new molecules or adducts that reduce the urinary supersaturation of cystine and consequently increasing the difficulty of crystallization of this substance. In these cases, avoiding the development of small crystals that initiate crystallization is very important for preventing the formation of all types of cystine calculi. In this case, the removal of small cystine crystals generated in the urine through increased water intake and physical activity can be a very important preventive measure.

## Figures and Tables

**Table 1:** Number of rough and smooth cystine renal stones generated by the 6 patients.

Patient	Rough Stones	Smooth Stones
Patient 1	2	2
Patient 2	2	1
Patient 3	2	2
Patient 4	2	0
Patient 5	2	7
Patient 6	1	7

## Data Availability

The data that support the findings of this study are available from the corresponding author [A.C.-B.] upon reasonable request.
